# Organisational Support, Interprofessional Collaboration and Patient Safety Perceptions Among Healthcare Professionals in Saudi Hospitals

**DOI:** 10.1155/jonm/6341795

**Published:** 2026-10-25

**Authors:** Romeo Jr Mostoles, Abdulhafith Alharbi

**Affiliations:** ^1^ Department of Psychiatric and Mental Health Nursing College of Nursing University of Hail Hail Saudi Arabia uoh.edu.sa

**Keywords:** interprofessional collaboration, mediation analysis, nursing management, organisational support, patient safety perceptions, Saudi Arabia

## Abstract

**Background:**

Organisational support and interprofessional collaboration (IPC) are each linked to patient safety (PS) culture, but the pathway connecting them has rarely been examined in Saudi Arabia, where hospitals are undergoing rapid workforce and structural transformation.

**Aims:**

To examine the associations among perceived organisational support (POS), IPC and patient safety perceptions (PSP) among healthcare professionals in Saudi hospitals, and to test whether IPC mediates the association between organisational support and PSP.

**Method:**

A cross‐sectional correlational study was conducted in six hospitals (five government, one private) in the Eastern and Northern regions of Saudi Arabia between 1 August and 31 October 2025. Hospital coordinators invited all eligible clinical staff through institutional channels, and 1328 healthcare professionals completed an anonymous online survey comprising the POS Scale, the 23‐item AITCS‐II (practitioner version) and the 33‐item Patient Safety Questionnaire from the WHO Patient Safety Curriculum Guide. Data were analysed with Pearson correlations and a bootstrapped single‐mediator path model in AMOS (5000 bias‐corrected [BC] resamples).

**Result:**

Organisational support was positively associated with IPC (*β* = 0.31, *p* < 0.001) and with PSP (*β* = 0.14, *p* < 0.001), whereas collaboration was negatively associated with PSP (*β* = −0.15, *p* < 0.001). The indirect association through collaboration was small and negative (*β* = −0.05, 95% CI [–0.07, −0.02]), indicating inconsistent (competitive) mediation; the total association remained positive but modest (*β* = 0.09, *p* < 0.001).

**Conclusion:**

POS is directly associated with more favourable PSP, but this association is partly offset by a small negative association operating through collaboration. Because the design is cross‐sectional and all constructs were self‐reported, the mechanisms underlying this pattern remain untested.

**Implications For Nursing Management:**

Nurse managers should strengthen visible organisational support (resources, recognition, responsive leadership) while recognising that increasing the frequency of interprofessional contact alone may not improve safety perceptions. Attention to the quality of collaborative processes, together with routine assessment of team climate and safety perceptions, offers a more targeted approach than expanding collaboration per se.

## 1. Introduction

Ensuring patient safety (PS) and optimising outcomes remain foundational objectives for healthcare systems worldwide. Interprofessional health teams (IHTs), composed of practitioners from diverse professions, have become central to delivering comprehensive, patient‐centred care, as emphasised by the World Health Organization (WHO). Effective interprofessional collaboration (IPC) characterised by coordinated communication, shared objectives and mutual trust enhances PS, reduces medical errors, improves treatment adherence and fosters a positive work environment for healthcare professionals [1, 2]. Numerous studies affirm that cohesive IHTs contribute significantly to improved safety culture, higher‐quality care and better clinical outcomes globally [3]. Despite this global consensus, the precise pathways through which organisational conditions and team collaboration jointly relate to safety perceptions remain inconsistently understood, especially in rapidly evolving healthcare systems.

In Saudi Arabia, IHTs represent a pivotal strategy for providing integrated healthcare services and optimising care delivery. Prior research highlights determinants such as communication, clarity of roles and trust as critical factors influencing IHT collaboration [4, 5]. Higher levels of team collaboration have been correlated with improved PS outcomes, enhanced teamwork and greater professional satisfaction [2]. However, the pathway linking organisational support to patient safety perceptions (PSP), and the extent to which team collaboration accounts for part of that association, remain understudied within the Saudi context. Recent WHO‐led initiatives and national safety frameworks emphasise the importance of interprofessional education, leadership and reporting systems to cultivate safety culture across Saudi healthcare institutions [1].

Organisational support has been proposed as an antecedent associated with team collaboration and, in turn, with PS perceptions—that is, organisational support is conceptualised as the predictor, collaboration as the mediator and PSP as the outcome. It encompasses institutional provisions such as adequate resources, supportive leadership, recognition, psychological safety and professional growth opportunities [6, 7]. Empirical evidence from Saudi healthcare facilities indicates that insufficient organisational support hinders effective teamwork, contributing to high staff turnover, burnout and diminished PS culture [5, 6]. Conversely, hospitals that demonstrate strong organisational backing through coaching, policy alignment and transparent communication report higher teamwork efficiency and better PS metrics [2, 7].

Organisational support also reinforces social capital within teams, fostering trust, collaboration and open dialogue that collectively advance PS [2]. Psychological safety cultivated through supportive institutional policies further enables team members to voice concerns and share information without fear of blame, reducing medical errors and fostering continuous quality improvement [3, 5]. This evidence underscores the necessity of integrating IPC frameworks with organisational support strategies tailored to Saudi Arabia’s healthcare system.

Although global studies affirm the importance of organisational support and IPC for PS improvement, research targeting the Saudi context has generally focused on immediate determinants like communication and role clarity without examining whether team collaboration operates as an intervening pathway between organisational support and PSP [4–6]. This gap is rooted in the unique features of the Saudi healthcare system, including its hierarchical structure, rapid expansion, administrative decentralisation and evolving regulatory frameworks [5] that pose challenges for applying international lessons locally. Previous Saudi studies have documented how insufficient organisational support is associated with staff turnover and burnout, whereas stronger support environments are associated with better teamwork and safety metrics [5–7]. However, the indirect associations linking organisational support, team collaboration and PSP have not been examined, and clarifying these patterns will contribute nuanced, context‐sensitive evidence for policy and practice in Saudi hospitals.

Specifically, whether IHT collaboration statistically accounts for part of the association between organisational support and PS perceptions in the Saudi context has yet to be empirically examined. Therefore, this study examined the direct association between organisational support and PS perceptions and the indirect association through IHT collaboration, using a cross‐sectional mediation model.

## 2. Methodology

### 2.1. Design

This study utilised a quantitative cross‐sectional correlational predictive design employing mediation analysis. The design was chosen to examine whether IHT collaboration mediates the relationship between perceived organisational support (POS) (independent variable) and PS perceptions (dependent variable) among healthcare professionals in Saudi Arabia. In the hypothesised model, organisational support was specified as the predictor, IPC as the mediator and PSP as the outcome (Figure 1).

**FIGURE 1:**
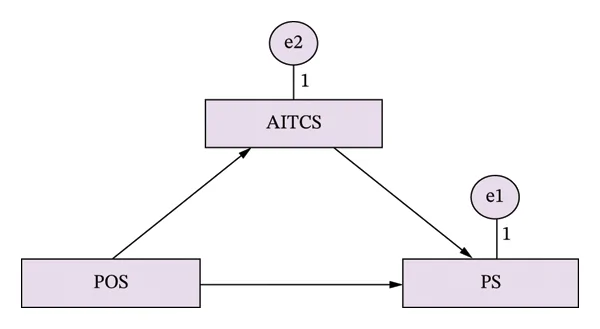
Mediation model of perceived organisational support, interprofessional collaboration and patient safety perceptions. Note. POS = perceived organisational support (POS scale total score); AITCS = interprofessional collaboration (AITCS‐II total score); PSP = patient safety perceptions (PSQ total score, reflecting self‐reported knowledge, attitudes and perceptions rather than objective safety outcomes). Standardised coefficients are reported in Table 5.

### 2.2. Population, Setting and Sampling

The target population comprised healthcare professionals providing direct patient care in hospitals located in the urban centres of the Eastern and Northern regions of Saudi Arabia. Professionals were eligible if they were (a) employed as a registered nurse, physician, medical technologist, physical therapist, or other licensed allied health professional; (b) currently working in a clinical role at a participating hospital; and (c) had been employed at the hospital for at least 6 months. Administrative staff without a clinical role and students or interns were not eligible.

A multistage, nonprobability sampling strategy was used. In the first stage, nine hospitals (five in the Eastern Region and four in the Northern Region) were purposively identified as candidate sites according to predefined criteria: geographical centrality, facility type and size and affiliation with the regional health cluster (Eastern Health Cluster or Hail Health Cluster) or, for private facilities, Ministry of Health licensure.

In the second stage, six hospitals (three per region) were purposively selected from these candidates on the basis of accessibility to the research team, size of the clinical workforce and administrative willingness to participate. The participating facilities were, in the Northern Region, Prince Abdulaziz Bin Musaed Hospital (government), the Maternity and Children Hospital (government) and the Cardiac and Diabetic Specialty Center (government); and, in the Eastern Region, King Fahd Specialist Hospital—Dammam (government), Saud Al‐Babtain Center for Cardiac Medicine and Surgery—Dammam (government) and Almoosa Specialist Hospital—Al‐Ahsa (private). In total, therefore, six facilities participated: three in each region; five government and one private; four general or specialist hospitals and two hospital‐based specialty centres. For brevity, all six are referred to as hospitals throughout.

In the third stage, a census (total population) invitation approach was used within each participating hospital: all healthcare professionals meeting the eligibility criteria were invited, and participation was voluntary; respondents therefore constitute a self‐selected (volunteer) sample rather than a probability sample. Invitations were distributed by designated hospital coordinators rather than by the research team (see Data Collection); because the number of professionals who received the invitation could not be determined, a response rate cannot be reported. A total of 1328 complete, valid questionnaires were received and retained for analysis; questionnaires with missing responses were excluded. Participants were classified as registered nurses, physicians, medical technologists, physical therapists and other healthcare professionals (pharmacists, respiratory therapists, laboratory technicians, radiology technologists, nutritionists and occupational therapists, combined because of small subgroup sizes).

### 2.3. Instrument

The questionnaire developed for this study was structured into four primary sections: a demographic profile, the POS Scale, the Assessment of Interprofessional Team Collaboration Scale‐II (AITCS‐II), practitioner version) and the Patient Safety Questionnaire (PSQ). The first section collected demographic and professional information, including age, gender, marital status, educational qualification, nationality, professional role and hospital area.

The scoring procedures recommended by the developers or validation authors were followed for all three instruments, and higher scores indicate more positive perceptions of each construct. Negatively worded items were reverse‐coded before totals were calculated. The POS Scale was administered in its published Arabic translation; the AITCS‐II was administered in its original English form; and the PSQ, originally developed for healthcare students, was reviewed for its applicability to practising professionals before use, as described below. No items were added, removed, or reworded in any instrument.

The POS Scale was created by Eisenberger et al. [8]. The scale was translated into Arabic by Mohammed Looti [9] to measure how much employees believe that their employer values them and is concerned with their welfare. All 17 items on this scale were answered using a 7‐point Likert scale, where 1 means ‘*strongly disagree*’ and 7 means ‘*strongly agree*’. Responses to negatively worded items were reversed prior to being added to provide a single total POS score ranging from 17 to 119. Consequently, higher total POS scores reflect an employee’s perception of receiving greater organisational support. Prior studies established this tool as reliable, reporting internal reliability (alpha coefficients) between 0.88 and 0.93 [9, 10].

#### 2.3.1. AITCS‐II

IPC was measured with the 23‐item AITCS‐II, practitioner version, developed by Orchard, Pederson, Read, Mahler and Laschinger [11]. The AITCS‐II is the revised and shortened form of the original 37‐item AITCS developed and validated by Orchard, King, Khalili and Bezzina [12]. In the revision study, Orchard et al. [11] re‐tested the original instrument in a new sample of practising health professionals, removed poorly performing items and reduced the scale to 23 items while retaining the three original subscales: partnership (8 items), cooperation (8 items) and coordination (7 items). Items are rated on a 5‐point Likert scale from 1 (*never*) to 5 (*always*). Item responses were summed to obtain a total score (range 23–115) and subscale scores were obtained by summing items within each subscale: partnership (8–40), cooperation (8–40) and coordination (7–35). Higher scores indicate stronger perceived IPC. The total score was used in the primary analyses and subscale scores for descriptive reporting.

In the revision and validation study, the AITCS‐II demonstrated a stable three‐factor structure with Cronbach’s alpha values of 0.936 (partnership), 0.927 (cooperation) and 0.898 (coordination) and 0.928 for the total scale [11]. Comparable reliability and factor structure have been reported in a subsequent cross‐cultural adaptation of the AITCS‐II for occupational health professionals in Sweden [13]. The instrument was administered in its original English form, which is the working language of the participating hospitals, and no items were added, removed, or reworded. In the present sample, Cronbach’s alpha for the total scale was 0.90.

#### 2.3.2. PSQ

PSP were assessed with the patient‐safety survey recommended in the WHO Patient Safety Curriculum Guide: Multiprofessional Edition [14], referred to here as the PSQ. The items originate from the survey developed by Madigosky et al. [15] and were adapted by Leung and Patil [16]. The 33 items are grouped into five domains: error and PS knowledge (7 items), safety of the healthcare system (6 items), personal influence over safety (7 items), personal attitudes towards PS (4 items) and safety at the workplace (9 items). Items are rated on a 5‐point Likert scale from 1 (*strongly disagree*) to 5 (*strongly agree*). After reverse‐coding negatively worded items, item scores were summed to yield domain scores and an overall total (possible range 33–165); higher scores indicate greater PS knowledge and more favourable attitudes and perceptions.

#### 2.3.3. Rationale for Use With Practising Professionals

Although the PSQ was first developed for students, its content is not student‐specific. The instrument is embedded in the WHO multiprofessional curriculum guide, whose stated scope covers all health professions [14], and its items address error knowledge, system safety, personal influence over safety, attitudes and workplace safety, all of which are experienced directly by clinicians in practice rather than only anticipated by learners. Consistent with this, the instrument has been applied without structural change to practising physicians in Egypt, a culturally and organisationally proximate Arab healthcare setting, where it showed acceptable internal consistency (*α* = 0.72) [17]. It has also been used in the Saudi context, among medical students [18], and in Iran [19], indicating that its content is interpretable in Middle Eastern healthcare cultures.

#### 2.3.4. Validity Evidence for the Present Population and Context

Because no version of the PSQ has been formally validated among practising professionals in Saudi Arabia, the following steps were taken and the following evidence is available from the present study.

#### 2.3.5. Content and Face Validity

Before data collection, an expert panel of 5 experienced Saudi healthcare providers (two nurse managers, two physicians and one quality/patient‐safety officer, each with more than 3 years of hospital experience) reviewed every item for relevance to hospital‐based practice in Saudi Arabia and for clarity of wording in English, the working language of the participating hospitals. The panel judged all 33 items to be applicable to practising professionals and no item required rewording, addition, or removal, since none of the items depend on student status. The questionnaire was then pilot‐tested with healthcare workers at a private hospital that was not among the six study sites. The pilot was qualitative, aimed at comprehension and acceptability; no problems with item understanding were identified and no changes were made. Formal content‐validity indexing and pilot reliability testing were not performed.

#### 2.3.6. Internal Consistency

In the present sample, Cronbach’s alpha for the 33‐item total was 0.89, higher than the value reported among Egyptian physicians [17] and indicating that the items functioned coherently as a set in this population.

#### 2.3.7. Internal Structure (Descriptive)

The five domain scores showed a differentiated profile (Table 1), with safety at the workplace highest and safety of the healthcare system lowest, and each domain used most of its possible range, indicating that the domains were not answered uniformly and that the instrument captured variation among practising respondents.

**TABLE 1:** Descriptive statistics of organisational support, interprofessional collaboration and patient safety perceptions.

Variables	Mean	SD	Minimum	Maximum
POS scale	70.4	11.2	34.0	112.0
Partnership	25.1	9.6	8.0	40.0
Cooperation	26.1	10.4	8.0	40.0
Coordination	22.7	8.5	7.0	35.0
AITCS‐II total	73.9	27.1	23.0	115.0
Error and patient safety knowledge	24.6	6.7	7.0	35.0
Safety of the healthcare system	17.3	2.1	9.0	25.0
Personal influence over safety	23.9	5.1	10.0	33.0
Personal attitudes towards patient safety	14.7	3.9	4.0	20.0
Safety at the workplace	31.2	6.0	13.0	41.0
PSQ total (patient safety perceptions)	111.7	17.1	53.0	145.0

*Note:* Higher scores indicate greater perceived organisational support, stronger interprofessional collaboration and more favourable patient safety knowledge, attitudes and perceptions (PSQ).

#### 2.3.8. Convergent and Nomological Evidence

Organisational‐support theory predicts that staff who perceive greater support hold more favourable safety perceptions [20, 21]. In the present sample, the PSQ total correlated positively with the POS Scale (*r* = 0.094, *p* < 0.001; Table 2), in the theoretically expected direction, and PSQ scores differed significantly across professional groups, gender and educational attainment (Table 3), showing that the instrument discriminates between subgroups of practising professionals. These associations are weak to modest and are reported as supporting, not definitive, evidence.

**TABLE 2:** Means, standard deviations and Pearson correlations among study variables (*N* = 1328).

Variable	*M*	SD	1	2	3
1. Perceived organisational support (POS)	70.4	11.2	(0.92)		
2. Interprofessional collaboration (AITCS‐II)	73.9	27.1	0.309^∗∗^	(0.90)	
3. Patient safety perceptions (PSP)	111.7	17.1	0.094^∗∗^	−0.10^∗∗∗^	(0.89)

*Note: N* = 1328. Pearson correlations (two‐tailed). Cronbach’s alpha on the diagonal in parentheses. Correlations are unadjusted zero‐order coefficients; the AITCS‐II–PSQ correlation is the unadjusted relationship between the mediator and the outcome.

^∗^ *p* < 0.01.

^∗∗∗^ *p* < 0.001.

**TABLE 3:** Comparison of POS, AITCS‐II and PSQ scores according to demographic characteristics (*N* = 1328).

Characteristic	Category	*N*	POS mean	POS SD	*p* value	AITCS‐II mean	AITCS‐II SD	*p* value	PSQ mean	PSQ SD	*p* value
Age, years	≤ 25	176	71.93	12.86	0.167	65.02	31.70	< 0.001	108.70	22.37	0.017
	26–30	172	70.16	11.85		77.19	26.17		113.81	20.00	
	≥ 31	980	70.23	10.71		74.93	26.04		111.86	15.34	

Gender	Female	1212	71.15	10.92	< 0.001	73.98	27.29	0.760	113.30	15.30	< 0.001
	Male	116	63.17	11.28		73.17	25.00		94.93	24.64	

Marital status	Single	420	70.78	2.95	0.462	72.01	26.65	0.083	115.40	14.04	< 0.001
	Married	908	70.30	0.26		74.78	27.25		109.98	18.12	

Educational attainment	Diploma	352	73.19	12.77	< 0.001	68.81	31.42	< 0.001	109.83	21.69	< 0.001
	Bachelor’s degree	500	71.72	10.41		77.55	25.55		112.20	10.36	
	Master’s‐level coursework, degree not completed	100	69.04	5.02		63.60	27.27		117.20	20.30	
	Master’s degree completed	292	66.27	11.11		75.42	23.55		111.77	14.68	
	Doctoral‐level coursework, degree not completed	48	70.50	9.63		77.50	16.64		128.25	9.30	
	PhD degree completed	36	63.67	6.10		84.67	24.85		85.00	23.48	

Nationality	Saudi	1192	70.47	10.90	0.814	72.22	26.89	< 0.001	111.87	15.47	0.284
	Non‐Saudi	136	70.24	13.40		88.68	24.20		110.21	27.68	

Profession	Registered nurse	576	70.28	7.81	< 0.001	72.06	28.40	< 0.001	110.41	16.41	< 0.001
	Medical technologist	136	66.88	9.80		69.00	29.98		114.53	13.46	
	Physical therapist	60	71.00	5.70		63.00	12.76		103.60	12.88	
	Physician	108	62.89	13.35		82.67	21.94		117.33	25.26	
	Other healthcare professionals	448	73.50	13.76		77.13	25.99		112.21	16.51	

Hospital area	Ward	316	70.65	10.39	0.002	72.01	32.05	0.363	112.65	22.91	0.177
	Special area (ER, OR, and DR)	260	68.32	10.77		74.43	20.97		112.75	12.06	
	Other	752	71.10	11.55		74.52	26.68		110.93	15.69	

*Note:* Independent‐samples *t*‐test (Welch’s correction applied where Levene’s test indicated unequal variances) for two‐category variables; one‐way ANOVA (with Games–Howell post hoc tests, selected because Levene’s test indicated unequal variances and group sizes were markedly unbalanced) for variables with three or more categories. Means and standard deviations are shown in separate columns. Significance set at *p* < 0.05.

#### 2.3.9. Use of the Total Score

The total score was used as the outcome in the primary analyses for three reasons. First, the study hypothesis concerned PSP as a single overarching construct, and a single‐mediator path model with one outcome was specified a priori; entering five correlated domain scores would have required five separate models and inflated the Type I error rate. Second, the five domains are conceptual facets of one orientation towards PS, spanning knowledge, attitudes and perceptions of the system and workplace, and the high internal consistency of the total (*α* = 0.89) is consistent with treating the 33 items as a single scale in this population. Third, a total score has been used in prior applications of the instrument, including among practising physicians [17]. Domain scores are reported descriptively (Table 1) so that the response profile across domains can be examined. Because the factor structure of the instrument was not examined in the present sample and measurement invariance across professional and language groups was not tested, the total score should be interpreted as a global index of PSP rather than as evidence of a unidimensional construct (see Limitations).

### 2.4. Data Collection and Ethical Consideration

Data were collected over a single 13‐week period from 1 August to 31 October 2025. Following ethical approval from the Institutional Review Board of the Hail Health Cluster (No. 2025‐100), which was accepted by the participating Eastern Region hospitals, and written institutional authorisation from each site, hospital management designated one coordinator per hospital. Each coordinator identified eligible healthcare professionals from institutional staff lists on the basis of job title and distributed the recruitment invitation, containing the study information sheet and the anonymous Google Forms link, through the institution’s official e‐mail system or other approved professional communication channels. Staff e‐mail addresses and contact lists were therefore held and used only by the hospital coordinators, who had access to them as part of their normal institutional roles; the research team did not receive, access or retain any staff e‐mail addresses or contact lists at any stage. One general reminder was circulated by the coordinators through the same channels approximately midway through the collection period; no individual follow‐up was undertaken. Coordinators were instructed by hospital management not to monitor participation, request evidence of completion, or follow up individually with staff, and they had no means of knowing who had responded because the survey collected no identifying information. The Google Form was configured not to collect e‐mail addresses or account information. The first screen of the form presented the information sheet, and participants indicated consent by selecting ‘I agree to participate’ before the questionnaire items were displayed; participants could withdraw at any time by closing the browser before submission. Because the findings reflect perceptions during this single collection window only, they should not be read as trends over time.

### 2.5. Data Analysis

Responses were exported from Google Forms into IBM SPSS Statistics version 28 and checked for missing values, out‐of‐range entries and outliers. Descriptive statistics summarised participant characteristics and scale scores. Cronbach’s alpha was computed for each scale in the present sample as a check of internal consistency (Table 2 diagonal). Group differences in POS, AITCS‐II and PSQ scores were examined with independent‐samples *t*‐tests (Welch’s correction where Levene’s test indicated unequal variances) and one‐way ANOVA with Games–Howell post hoc tests. Bivariate associations among the three study variables were examined with Pearson correlations before the mediation model was fitted. Normality, linearity and homoscedasticity were checked with histograms, Q–Q plots and residual scatterplots. Statistical significance was set at *p* < 0.05 (two‐tailed) with 95% confidence intervals. A post hoc power analysis (G∗Power 3.1) indicated that *N* = 1328 provided > 99% power to detect a correlation of *r* = 0.10 at *α* = 0.05.

### 2.6. Mediation Analysis

The hypothesised model was tested as a single‐mediator path model in IBM SPSS AMOS version 32, with POS (POS total score) as the predictor, IPC (AITCS‐II total score) as the mediator and PSP (PSQ total score) as the outcome. All three constructs were entered as observed (manifest) composite scores rather than latent variables; therefore, the estimates are not corrected for measurement error. Paths were specified from POS to AITCS‐II (Path a), from AITCS‐II to PSQ (Path b) and from POS to PSQ (Path c′), with independent residual terms for the mediator and outcome. Because all possible paths among the three variables were estimated, the model was saturated (df = 0) and conventional fit indices were not applicable; model evaluation therefore focused on parameter estimates and their confidence intervals.

Parameters were estimated using maximum likelihood. The indirect effect (a × b) and total effect (c′ + a × b) were obtained from the AMOS standardised and unstandardised indirect/total effects output, and their significance was assessed with BC percentile bootstrap confidence intervals based on 5000 bootstrap resamples with replacement at the 95% confidence level (random seed = 1). An effect was considered significant when the BC interval excluded zero. Both unstandardised (B) and standardised (*β*) estimates are reported. Given the opposite signs of the direct and indirect paths, mediation was classified following Zhao, Lynch and Chen [22] as complementary or competitive (inconsistent) mediation. Throughout, the terms direct, indirect and total ‘effects’ are used in their conventional path‐analytic sense to denote model‐implied associations; because the design is cross‐sectional, they do not represent causal effects.

#### 2.6.1. Covariates

The mediation model was prespecified as an unadjusted three‐variable model, and no demographic or professional covariates were entered. This decision reflected three considerations. First, the study hypotheses concerned the overall and mediated associations among the three focal constructs in the healthcare workforce as a whole, not their conditional associations within demographic strata. Second, the demographic variables were collected as categorical variables with markedly unbalanced groups (e.g., 116 males versus 1212 females; 36 doctoral‐degree holders versus 500 bachelor’s‐degree holders), so entering them as dummy‐coded covariates would have produced unstable estimates for the small categories. Third, several demographic variables were associated with the mediator or the outcome (Table 3) and could plausibly act as confounders, but a cross‐sectional design does not allow their causal status (confounder versus collider or downstream consequence) to be determined, and conditioning on variables of uncertain status can introduce rather than remove bias. The unadjusted estimates may therefore be subject to confounding by gender, profession, nationality or education. To allow readers to judge the likely direction of any such confounding, the demographic comparisons on all three study variables are reported in full in Table 3, and this issue is acknowledged in the Limitations.

#### 2.6.2. Clustering by Hospital

Participants were nested within six hospitals, and responses within a hospital may be more similar than responses across hospitals. Hospital identity was deliberately not recorded on the anonymous questionnaire to protect participant confidentiality, because some professional subgroups within a single site were small enough to be potentially identifiable. Consequently, neither hospital nor region could be entered as a clustering variable; the intraclass correlation could not be estimated and multilevel or cluster‐robust modelling was not possible. All analyses therefore treat responses as independent. If hospital‐level variance in the study variables exists, standard errors are likely to be underestimated and confidence intervals too narrow. It should also be noted that with only six clusters, a multilevel model would itself have had limited power to estimate between‐hospital variance reliably. This constraint is acknowledged in the Limitations.

## 3. Results

Throughout the Results, ‘patient safety perceptions’ (PSP) refers to the PSQ total score, a self‐report index of PS knowledge, attitudes and perceptions; no objective safety outcomes were measured.

Table 4 presents the sociodemographic characteristics of the participants. Most respondents were aged 31 years and above (73.8%), female (91.3%), married (68.4%) and Saudi nationals (89.8%). Regarding educational attainment, 37.7% held a bachelor’s degree and 26.5% held a diploma. In addition, 7.5% had completed master’s‐level coursework but had not completed the degree, 22.0% held a master’s degree, 3.6% had completed doctoral‐level coursework but had not completed the degree and 2.7% held a doctoral degree. Registered nurses constituted the largest professional group (43.4%), followed by other healthcare professionals (33.7%), medical technologists (10.2%) and physicians (8.1%). More than half of the participants (56.6%) worked in other hospital areas, while 23.8% were assigned to general wards and 19.6% worked in special areas, including the emergency room (ER), operating room (OR) and delivery room (DR).

**TABLE 4:** Sociodemographic characteristics of participants (*N* = 1328).

Variable		Count	(%)
Age	25 years old and below	176	(13.3)
	26–30 years	172	(13.0)
	31 years old and above	980	(73.8)

Gender	Female	1212	(91.3)
	Male	116	(8.7)

Marital status	Single	420	(31.6)
	Married	908	(68.4)

Educational background	Diploma	352	(26.5)
	Bachelor’s degree	500	(37.7)
	Master’s‐level coursework, degree not completed	100	(7.5)
	Master’s Degree completed	292	(22.0)
	Doctoral‐level coursework, degree not completed	48	(3.6)
	PhD Degree completed	36	(2.7)

Nationality	Saudi	1192	(89.8)
	Non‐Saudi	136	(10.2)

Profession	Registered nurse	576	(43.4)
	Medical technologist	136	(10.2)
	Physical therapist	60	(4.5)
	Physician	108	(8.1)
	Other healthcare professionals	448	(33.7)

Hospital area	Ward	316	(23.8)
	Special area (ER, OR, DR)	260	(19.6)
	Other	752	(56.6)

Table 1 presents the descriptive statistics for the main study variables. The mean score for POS was 70.4 (SD = 11.2), indicating a moderately high level of perceived support among participants. Within the AITCS‐II, cooperation had the highest mean score (*M* = 26.1, SD = 10.4), followed by partnership (*M* = 25.1, SD = 9.6) and coordination (*M* = 22.7, SD = 8.5). The AITCS‐II total had a mean of 73.9 (SD = 27.1), reflecting moderate collaboration levels. Among the PSQ subdomains, safety at the workplace had the highest mean (*M* = 31.2, SD = 6.0), whereas safety of the healthcare system was the lowest (*M* = 17.3, SD = 2.1). The PSQ total score averaged 111.7 (SD = 17.1), indicating generally favourable PS knowledge, attitudes and perceptions among healthcare professionals.

Table 2 presents the complete bivariate correlation matrix. POS was positively correlated with IPC (*r* = 0.309, *p* < 0.001) and weakly with PSP (*r* = 0.094, *p* < 0.001). IPC was weakly and negatively correlated with PSP (*r* = −0.105, *p* < 0.001). The negative collaboration–safety association was therefore present in the unadjusted data and is not an artefact of conditioning on POS in the mediation model; the standardised total effect in Table 5 (*β* = 0.094) equals the zero‐order POS–PSQ correlation, as expected in a saturated model.

**TABLE 5:** Direct, indirect and total path estimates (associations) among perceived organisational support, interprofessional collaboration and patient safety perceptions (*N* = 1328).

Pathway	Estimate B (*β*)	SE	CR/Z	95% BC bootstrap CI (unstandardised)	*p* value
Direct paths					
POS ⟶ AITCS‐II	**0.749 (0.309)**	**0.063**	**11.85**	**0.648 to 0.839**	**< 0.001^∗^**
AITCS‐II ⟶ PSP	**−0.094 (−0.148)**	**0.018**	**−5.21**	**−0.138 to −0.047**	**< 0.001^∗^**
POS ⟶ PSP	**0.214 (0.139)**	**0.044**	**4.9**	**0.132 to 0.300**	**< 0.001** ^∗^
Indirect path					
POS ⟶ AITCS‐II ⟶ PSP	**−0.070 (−0.046)**	**0.018**	**−3.89**	**−0.104 to −0.036**	**< 0.001^∗^**
Total association					
POS ⟶ PSP (total)	**0.143 (0.094)**	**0.038**	**3.76**	**0.072 to 0.219**	**< 0.001^∗^**

*Note:* Bold and asterisk indicate significant results (*p* < 0.05). Unstandardised estimates (B) with standardised coefficients (*β*) in parentheses. Bias‐corrected percentile bootstrap, 5000 resamples. Model is saturated (df = 0); fit indices not applicable. PSP = patient safety perceptions (PSQ total score).

Table 3 compares POS, AITCS‐II and PSQ scores across demographic groups. POS differed significantly by gender (*p* < 0.001), educational attainment (*p* < 0.001), profession (*p* < 0.001) and hospital area (*p* = 0.002), but not by age, marital status or nationality. Females reported higher POS (*M* = 71.15, SD = 10.92) than males (*M* = 63.17, SD = 11.28). Participants with a diploma reported the highest POS (*M* = 73.19, SD = 12.77) and those with a completed doctoral degree the lowest (*M* = 63.67, SD = 6.10). Among professions, ‘other healthcare professionals’ reported the highest POS (*M* = 73.50, SD = 13.76) and physicians the lowest (*M* = 62.89, SD = 13.35). Staff in other hospital areas (*M* = 71.10, SD = 11.55) and general wards (*M* = 70.65, SD = 10.39) reported higher POS than staff in special areas (ER, OR, DR; *M* = 68.32, SD = 10.77).

AITCS‐II scores differed significantly by age (*p* < 0.001), educational attainment (*p* < 0.001), nationality (*p* < 0.001) and profession (*p* < 0.001), but not by gender, marital status or hospital area. Participants aged 26–30 years reported the highest collaboration scores (*M* = 77.19, SD = 26.17) and those aged ≤ 25 years the lowest (*M* = 65.02, SD = 31.70). Non‐Saudi participants (*M* = 88.68, SD = 24.20) scored higher than Saudi participants (*M* = 72.22, SD = 26.89). Participants with a completed doctoral degree had the highest scores (*M* = 84.67, SD = 24.85) and those with incomplete master’s‐level coursework the lowest (*M* = 63.60, SD = 27.27). Physicians reported the highest collaboration scores (*M* = 82.67, SD = 21.94) and physical therapists the lowest (*M* = 63.00, SD = 12.76).

PSQ scores differed significantly by age (*p* = 0.017), gender (*p* < 0.001), marital status (*p* < 0.001), educational attainment (*p* < 0.001) and profession (*p* < 0.001), but not by nationality or hospital area. Participants aged 26–30 years had the highest PSQ scores (*M* = 113.81, SD = 20.00). Females (*M* = 113.30, SD = 15.30) scored higher than males (*M* = 94.93, SD = 24.64), and single participants (*M* = 115.40, SD = 14.04) scored higher than married participants (*M* = 109.98, SD = 18.12). Participants with incomplete doctoral‐level coursework reported the highest PSQ scores (*M* = 128.25, SD = 9.30) and those with a completed doctoral degree the lowest (*M* = 85.00, SD = 23.48). Physicians had the highest PSQ scores (*M* = 117.33, SD = 25.26) and physical therapists the lowest (*M* = 103.60, SD = 12.88).

Figure 1 illustrates the cross‐sectional mediation model estimated in AMOS. Standardised path coefficients indicate that POS was positively associated with both IPC (*β* = 0.31, *p* < 0.001) and PSP (PSP; *β* = 0.14, *p* < 0.001). In contrast, IPC was negatively associated with PSP (*β* = −0.15, *p* < 0.001). The indirect association between POS and PSP through IPC was negative (*β* = −0.05, 95% CI [–0.07, −0.02], *p* < 0.001), a pattern consistent with statistical suppression.

Table 5 presents the direct, indirect and total path estimates (*N* = 1328). POS was positively associated with IPC (*β* = 0.31, *p* < 0.001) and with PSP (*β* = 0.14, *p* < 0.001), indicating that higher organisational support was directly associated with both stronger collaboration and more favourable PSP. However, IPC was negatively associated with PSP (*β* = −0.15, *p* < 0.001), and the indirect association between POS and PSP through IPC was negative (*β* = −0.05, 95% CI [–0.07, −0.02], *p* < 0.001), indicating inconsistent (competitive) mediation, in which the direct and indirect associations have opposite signs [22, 23]. Consistent with this, the unadjusted correlation between collaboration and safety perceptions was also negative (*r* = −0.10). The total association between POS and PSP remained positive though modest (*β* = 0.09, *p* < 0.001). Thus, POS retained a positive direct association with PSP even though the indirect association through IPC was in the opposite direction.

Direct, indirect and total effects refer to standardised path estimates from a cross‐sectional structural model and do not imply causal effects.

## 4. Discussion

### 4.1. Organisational Support and IPC

The positive association between POS and IPC among healthcare professionals in Saudi hospitals is consistent with the view that organisational backing, characterised by engaged leadership, recognition and sufficient resources, provides conditions under which interprofessional teamwork can develop. The present study did not measure intervening processes such as psychological safety, trust or communication quality, so the pathway linking support to collaboration cannot be specified from these data; the wider literature, however, identifies these as plausible links.

Extensive reviews consistently show that organisational support, including visible leadership involvement and team recognition, facilitates IPC, trust and team cohesion in healthcare settings [24, 25]. Integrative reviews further confirm that initiatives promoting interprofessional education and communication yield measurable improvements in collaborative behaviours and clinical outcomes [25].

In the Saudi context, targeted interventions such as regular interdisciplinary meetings and structured recognition programs have proven effective in addressing local cultural and structural barriers to collaboration [26, 27]. However, some meta‐analyses indicate that the impact of organisational support on patient outcomes is moderate or varies depending on institutional context, highlighting the importance of organisational culture and resource availability [28–30].

These findings point to organisational support as a modifiable, manager‐level lever; specific recommendations are set out in the Implications for Nursing Management section. Continuous, data‐driven evaluation is needed to determine whether such interventions translate into improvements in safety perceptions and, ultimately, in patient care quality.

### 4.2. Organisational Support and PSP

The results show a significant positive association between organisational support and PSP. Healthcare professionals who perceived stronger organisational support, including access to resources, recognition and effective leadership, reported more favourable PS knowledge and attitudes. The wider literature suggests that such support is associated with stronger safety culture and teamwork, although perceptions are only one of many determinants of actual safety outcomes, which were not measured here.

Systematic reviews and meta‐analytic evidence reveal that positive work environments, robust systemic support and strong organisational commitment are strongly linked to improved employee safety behaviours and PS perceptions across healthcare settings; however, the strength of these associations varies by region and industry [20, 21]. Recent studies further show that effective teamwork, well‐established safety culture and optimal nurse staffing together with advanced technology were consistently associated with fewer errors and superior safety compliance [31, 32].

Nevertheless, the relationship between organisational support and PSP is multifactorial. Factors such as burnout, workload, staffing and workplace stress have been associated with both organisational support and safety perceptions in other studies [32–34]; because they were not measured in the present study, their contribution here remains unknown, but the wider literature suggests that addressing them may complement organisational‐support initiatives [35, 36]. High levels of burnout and workplace stress may further reduce the effectiveness of organisational strategies, highlighting the need for integrated approaches that address both workforce well‐being and safety. Additionally, research suggests the relationship is bidirectional and context‐dependent, with organisational support and safety climate influencing each other over time [37, 38].

Because burnout, staffing and psychosocial conditions were not measured here, recommendations arising from this association are confined to the support behaviours captured by the POS Scale (see Implications for Nursing Management). Within that scope, the relevant behaviours are recognition of contributions, responsiveness to staff concerns and advocacy for the resources staff needs to work safely. Complementary attention to workforce well‐being and communication climate, informed by local assessment rather than by the present findings alone, may help sustain favourable safety perceptions.

### 4.3. IPC and PSP

Contrary to expectations, IPC was negatively associated with PSQ scores (*β* = −0.15) once organisational support was accounted for. This diverges from the literature, which generally reports that stronger IPC accompanies better safety culture and outcomes. In German hospitals, higher IPC scores were associated with more favourable PSP [39], and effective physician–nurse collaboration has been linked to fewer safety incidents and a better safety climate where leadership and a nonpunitive culture are present [40].

However, the relationship between collaboration and safety perceptions may be more complex than a simple positive association [41]. Contextual factors that could shape this relationship, such as team training, communication structures or professional hierarchies, have been described in other settings [42] but were not assessed in the present study, and no inference about them is drawn here.

Any recommendation regarding trust, accountability, or team culture would extend beyond what was measured here and is offered in the Implications section only as a direction for local inquiry, aligned where appropriate with Saudi Vision 2030 health‐sector reforms.

### 4.4. Inconsistent Mediation: Interpretation and Alternative Explanations

The indirect association of organisational support with PSP through IPC was negative, while the direct association was positive. This inconsistent (competitive) mediation pattern arises statistically because collaboration scores were weakly and negatively correlated with safety‐perception scores (*r* = −0.105), an association that accounts for a small share of variance and should be interpreted cautiously.

Because this study measured only the three focal constructs, the reasons for the negative association cannot be determined from these data. Several explanations, none of which we tested, merit consideration. First, the PSQ captures self‐rated knowledge and attitudes rather than teamwork quality; professionals who collaborate more extensively may be more exposed to system weaknesses and therefore more critical in appraising safety, so that the negative association could reflect heightened awareness rather than poorer safety. Second, the AITCS‐II measures the frequency of collaborative behaviours rather than their quality; the literature distinguishes structured, cognitively oriented teamwork, which is consistently associated with safer care [43], from unstructured interaction that does not confer the same benefits [44]. Third, the association may be confounded by group characteristics that were measured but not entered as covariates; for example, profession and nationality were related to collaboration scores in this sample (Table 3). Because the mediation model was prespecified as unadjusted, these subgroup differences cannot be ruled out as partial explanations for the negative indirect path, and the pattern should be interpreted as a descriptive, population‐level association rather than a demographically adjusted estimate. Fourth, common‐method variance and the predominantly female, nurse‐heavy sample may have influenced the observed pattern. Finally, qualitative work on IPC has described professional hierarchies and role boundaries as factors that can shape how increased interaction is experienced [42]; however, hierarchy, power distance, professional silos, interprofessional conflict and diffusion of responsibility were not measured here, and this remains one of several untested possibilities rather than a finding of the present study.

The practical implication is modest and specific: fostering more frequent collaboration alone may not be sufficient to improve safety perceptions, and organisations should evaluate the quality of collaborative processes alongside their frequency. Future research should measure candidate mechanisms directly, using validated instruments for team climate, psychological safety and role clarity, complemented by qualitative or observational methods, and should test whether the negative association replicates in other samples and with objective safety indicators.

### 4.5. Implications for Nursing Management

Nurse managers occupy the organisational level at which perceived support is most directly shaped and at which IPC is enacted day to day. Organisational‐support theory holds that employees personify the organisation through the actions of their immediate supervisors [8, 10], so the support behaviours measured by the POS Scale are, for most frontline staff, experienced largely through unit‐level management. Registered nurses formed the largest professional group in this study (43.4%), and because nurse managers typically coordinate the interprofessional teams in which the remaining professions work, the findings are relevant to nursing management across the whole sample. All recommendations below are offered as associations to be acted on cautiously, since the cross‐sectional, self‐report design does not support causal claims.

First, POS was positively associated with both IPC and PSP. Nurse managers are the most visible source of organisational support for frontline staff, and the behaviours captured by the POS Scale—recognising contributions, responding to concerns, ensuring adequate resources and showing regard for staff well‐being—fall largely within their sphere of influence. Embedding these behaviours into routine practice (e.g., structured recognition during shift handovers, transparent follow‐up on staff‐raised concerns and advocacy for staffing and equipment at the unit level) requires no new instruments or programmes and, on the present data, is associated with more favourable collaboration and safety perceptions.

Second, the small negative association between collaboration frequency and safety perceptions cautions nurse managers against assuming that more interprofessional contact is, by itself, beneficial. The AITCS‐II measures how often collaborative behaviours occur, not their quality. Nurse managers should therefore attend to the structure and psychological safety of interprofessional interactions—such as using standardised communication tools during rounds and handovers, clarifying role boundaries and accountability for safety tasks and ensuring that nurses′ safety concerns raised in interprofessional settings are acted upon—rather than simply increasing the number of joint meetings or rounds. Structured team‐training programmes such as TeamSTEPPS, which have been evaluated in hospital wards [44], offer a ready‐made framework for this shift from frequency to quality. Whether such quality‐focused approaches alter the association observed here is a question for future evaluation.

Third, the exploratory subgroup comparisons in Table 3 point to groups that may warrant particular attention, although pairwise differences should be interpreted descriptively. Perceived support was lower among staff in specialised areas (ER, OR, and DR) than in general wards, and lower among physicians than among nurses, suggesting that unit‐level support strategies should be tailored rather than uniform. Male staff and physical therapists reported less favourable safety perceptions, and Saudi staff reported lower collaboration scores than non‐Saudi colleagues; nurse managers leading mixed‐nationality, multiprofessional teams should be alert to such differences and consider whether orientation, mentoring, or team‐building activities are reaching all subgroups equitably.

Fourth, the study supports routine, unit‐level monitoring. Brief, validated measures administered periodically (e.g., the POS Scale, a team‐climate or psychological‐safety measure that captures the quality rather than the frequency of collaboration, and a short safety‐climate measure at 6‐ to 12‐month intervals) would allow nurse managers to track whether support initiatives are accompanied by changes in staff perceptions, and to identify units where collaboration and safety perceptions diverge. Such data can also strengthen the case nurse managers make to senior leadership for resources.

Fifth, the findings have implications for how nurse managers themselves are prepared and supported. If POS is transmitted largely through managerial behaviour, then nurse‐manager development programmes should include explicit training in the support behaviours captured by the POS Scale and in facilitating structured interprofessional communication, and senior nursing leadership should ensure that unit managers have the authority and resources to act on staff concerns. Nurse managers who are themselves unsupported are unlikely to be perceived as a reliable source of support by their teams.

Sixth and finally, because the mechanisms underlying the negative collaboration–safety association were not measured, nurse managers should treat these findings as a prompt for local inquiry—through unit debriefings, staff forums, or focus groups—rather than as a prescription. Insights from such inquiry can be used to co‐design collaboration practices with the interprofessional team and to inform hospital‐level policy within the framework of Saudi Vision 2030 health‐sector transformation.

### 4.6. Limitations of the Study

This study has several limitations. First, the cross‐sectional design precludes causal inference; all direct, indirect and total effects are cross‐sectional associations, and the inconsistent mediation pattern should not be read as evidence that collaboration reduces safety perceptions. Second, all constructs were self‐reported by the same respondents at the same time, so common‐method variance may have inflated or distorted the observed associations. Third, PS was operationalised as self‐reported knowledge, attitudes and perceptions rather than objective indicators such as incident reports or adverse‐event rates; the findings should not be interpreted as evidence of effects on patient outcomes. Fourth, the study measured only the three focal constructs; candidate explanatory mechanisms discussed in the literature (team climate, psychological safety, professional hierarchy, role clarity) were not assessed, so the reasons for the observed pattern remain undetermined. Fifth, the mediation model was not adjusted for participant characteristics. This was a deliberate a priori decision (see Methods), but it means that the unadjusted paths, including the negative collaboration–safety path, may be partly confounded by gender, profession, nationality or education, each of which differed on at least one study variable (Table 3). Because the model was not re‐estimated with covariates, the robustness of the inconsistent‐mediation pattern to demographic adjustment remains untested and should be examined in future studies with larger and more balanced subgroups. Sixth, hospital identity was not recorded in the anonymised dataset; hospital‐level clustering therefore could not be modelled, and standard errors may be underestimated if hospital‐level variance exists. For the same reason, although five of the six hospitals were government facilities and one was private, no sector‐based comparison was possible and the findings primarily reflect the public hospital context. Seventh, the multistage nonprobability design (purposive selection of hospitals and voluntary participation of invited staff) introduces selection and self‐selection bias; professionals with stronger views on organisational support or teamwork may have been more inclined to respond, and because invitations were distributed by hospital coordinators, the response rate could not be determined. Eighth, the sample was predominantly female (91.3%) and nurse‐heavy (43.4%); because females reported higher POS and PSQ scores than males, the pattern of associations may not generalise to settings with different workforce composition. Finally, the PSQ has not been formally validated for practising professionals in Saudi Arabia. Its suitability was established through expert‐panel content review, a qualitative pilot, high internal consistency and theoretically expected associations with organisational support and demographic groups, rather than through content‐validity indexing, confirmatory factor analysis, or tests of measurement invariance across professional and language groups; the same applies to the AITCS‐II, which was administered in English to a workforce for whom English is a second language for many. The total PSQ score should therefore be read as a global index whose internal structure in this population remains to be confirmed.

## 5. Conclusion

This study shows that POS is associated with stronger IPC and more favourable PS knowledge and attitudes among healthcare professionals in Saudi hospitals. Mediation analysis identified a small negative indirect association through collaboration, the mechanisms of which were not measured and require further study. For nursing management, the findings direct attention to the everyday support behaviours of unit managers and to the quality, rather than the frequency, of IPC. Integrated organisational and interprofessional strategies, with ongoing evaluation, may help strengthen safety perceptions; longitudinal research linking these perceptions to objective safety indicators is needed before conclusions about patient outcomes can be drawn.

## Author Contributions

Abdulhafith Alharbi and Romeo Jr Mostoles conceptualised and wrote the draft of the study; Abdulhafith Alharbi performed the analysis and interpretation of the data; Romeo Jr Mostoles performed data gathering and wrote the manuscript; Abdulhafith Alharbi and Romeo Jr Mostoles wrote the final draft of the manuscript.

## Funding

This study did not receive funding from agency or any institution.

## Ethics Statement

Ethical approval was granted by the Institutional Review Board, Hail Health Cluster, Hail Region, Kingdom of Saudi Arabia (2025‐100). The research team confirmed that all methods were performed in accordance with the relevant guidelines and regulations. Informed consent was obtained from all the subjects.

## Consent

The authors have nothing to report.

## Conflicts of Interest

The authors declare no conflicts of interest.

## Data Availability

The datasets used and/or analysed during the current study are available from the corresponding author upon reasonable request.
